# The Network Model of Depression as a Basis for New Therapeutic Strategies for Treating Major Depressive Disorder in Parkinson’s Disease

**DOI:** 10.3389/fnhum.2016.00161

**Published:** 2016-04-22

**Authors:** Kevin D’Ostilio, Gaëtan Garraux

**Affiliations:** ^1^Movere Group, Cyclotron Research Center, University of LiegeLiege, Belgium; ^2^Department of Neurology, University Hospital CenterLiege, Belgium

**Keywords:** Parkinson’s disease, neurostimulation, depression, cognitive and behavioral therapy, transcranial magnetic stimulation

## Abstract

The high prevalence of major depressive disorder in people with Parkinson’s disease (PD), its negative impact on health-related quality of life and the low response rate to conventional pharmacological therapies call to seek innovative treatments. Here, we review the new approaches for treating major depressive disorder in patients with PD within the framework of the network model of depression. According to this model, major depressive disorder reflects maladaptive neuronal plasticity. Non-invasive brain stimulation (NIBS) using high frequency repetitive transcranial magnetic stimulation (rTMS) over the prefrontal cortex has been proposed as a feasible and effective strategy with minimal risk. The neurobiological basis of its therapeutic effect may involve neuroplastic modifications in limbic and cognitive networks. However, the way this networks reorganize might be strongly influenced by the environment. To address this issue, we propose a combined strategy that includes NIBS together with cognitive and behavioral interventions.

## Major Depressive Disorder in PD

### Prevalence and Features

Major depressive disorder is a widely distributed chronic condition. While 1-year prevalence estimates of this entity round 5–10%, the lifetime prevalence reaches up to 15–20%. Moreover, major depressive disorder is characterized by high relapsing rates: 22–50% of patients suffer recurrent episodes within 6 months after recovery (World Health Organization, 2014). It is associated with compromised quality of life, increased health care costs, and greater risk for a widespread medical conditions, particularly coronary heart disease.

Major depressive disorder is common in Parkinson’s disease (PD), a progressively debilitating neurological disorder affecting over 1 million individuals in the United States and approximately 6 millions worldwide. PD is considered as a movement disorder characterized by four cardinal motor symptoms: tremor, muscle rigidity, motor slowness and postural instability. However, most patients do also experience non-motor manifestations (Chaudhuri et al., 2006) such as cognitive or emotional disturbances that significantly contribute to their disability (Weintraub et al., 2004).

The prevalence of major depressive disorder in patients with PD (PD-dep) has not been well established. The average prevalence of clinically significant depressive symptoms was calculated to be close to 35% (Reijnders et al., 2008). However, estimates may vary according to different factors such as age and disease stage. High prevalence was reported in patients with younger age of diagnosis, peaking at onset or in the advanced stages (Schrag et al., 2003).

PD-dep has been speculated to have some distinctive features when compared with major depressive disorder in non-PD patients, including relatively high levels of anxiety, preservation of short-term memory and no association with severity of motor symptoms. In addition, irritability, sadness, dysphoria, pessimism and suicide ideation are more common in PD-dep whereas feelings of failure, self-blame, guilt and real suicide are less frequent (Burn, 2002).

Major depressive disorder has been shown to lead to increased disability in patients with PD, affecting activities of daily living, health-related quality of life, and functional status (Ravina et al., 2007). Indeed, some of the daily life impairments are strongly related to the inability to overcome depressive symptoms (Weintraub et al., 2004). In addition, PD-dep negatively impacts motor and cognitive abilities (Marsh, 2013). However, despite their major repercussion, affective disturbances in PD have received less attention compared to motor and cognitive dysfunctions.

## The Biochemical Model of Depression in PD

The etiology of PD-dep is not clear. According to the biochemical model, PD-dep would be secondary to neurobiological changes that result from the degenerative process, namely a decrease in monoamine levels (Mayeux et al., 1984; Chan-Palay and Asan, 1989; Remy et al., 2005).

The hallmark of PD is a loss of dopaminergic neurons in the ventrolateral tiers of the substantia nigra *pars compacta* in midbrain (Fearnley and Lees, 1991) leading to a deficiency of dopaminergic terminals in the posterior “motor” aspects of the striatum (Kish et al., 1988). This results into motor disturbances such as movement slowness and increased muscle tone. The midbrain also contains a distinct cluster of dopaminergic neurons lying in the ventral tegmental area (VTA) that may be affected in PD. These neurons project to cognitive and limbic areas. The targets of the mesocortical “cognitive” pathway are the anterior cingulate, entorhinal and prefrontal cortices whereas the mesolimbic “reward” pathway is directed to to the ventral striatum, bed nucleus of the stria terminalis, hippocampus, amygdala and septum (Dunlop and Nemeroff, 2007). Therefore, by affecting these nodes of the limbic and cognitive systems, dopaminergic deficiency is likely to be involved in PD-dep pathophysiology. This is supported by some clinical observations: for instance, the occurrence of depressive symptoms is sometimes specifically associated with off dopaminergic states whereas dopamine agonists such as pramipexole (i.e., a D2/D3 receptor agonist) have shown antidepressant effects regardless of motor improvement (Rektorová et al., 2003).

However, the assumption that basal ganglia-cortical loops are organized into distinct functional zones has been challenged. A relationship between the motor and the limbic networks is supported by deep brain stimulation (DBS) studies. Thus, although high-frequency electrical stimulation into the subthalamic nucleus (STN) is able to improve motor symptoms in advanced PD, it can also either improve or induce depressive symptoms (Takeshita et al., 2005; Temel et al., 2009). Additionally, information processing in brain networks depends on the pattern of activity across different frequency bands. While beta activity may serve as a channel for motor processing in PD, existing evidence supports the hypothesis that emotion processing is associated with alpha activity in basal ganglia cortical networks. Indeed, alpha activity is modulated by dopaminergic therapy and emotional state in PD (Kühn et al., 2005; Huebl et al., 2014) and is also correlated with symptom severity in severely depressed patients without PD (Neumann et al., 2014).

The relationship between dopamine signaling, synaptic plasticity and depression is complex. In their review, Lavergne and Jay (2010) presented evidence suggesting that major depressive disorder is associated with decreased dopamine signaling and decreased synaptic plasticity in several brain regions, especially in the prefrontal cortex. In addition, dopamine release in the prefrontal cortex was observed in response to most pharmacological treatments, independently of their mechanism of action. They proposed a model where major depressive disorder is a state of dopaminergic-induced synaptic depression in the frontal cortex while optimal dopamine transmission would restore synaptic potentiation. According to the model, this relationship is not linear but rather follows an inverted U curve: too little or too much dopamine signaling in the frontal cortex would be associated to a state of clinical and synaptic depression. In PD, there is evidence supporting an inverted U shape relationship between frontal lobe functions and dopaminergic states regarding cognition (Cools et al., 2001). However, to the best of our knowledge, this model has never been explicitly tested for PD-dep.

In PD, neurodegenerative processes not only affect dopamine neurotransmission but also other monoaminergic neurons including noradrenergic and serotonergic systems, which may also play a role in the pathophysiology (Lang and Lozano, 1998). Supporting this view, a positron emission tomography (PET) study using a noradrenergic radiotracer found that PD-dep was associated with a reduced uptake in the limbic system (Remy et al., 2005). In addition, serotonergic neuron loss within the raphe nuclei has also been demonstrated in PD (Paulus and Jellinger, 1991).

However, a direct causal relationship between major depressive disorder and disrupted monoamine neurotransmission has been questionned since serotonin and catecholamine depletion do not affect mood in healthy subjects (Ruhé et al., 2007). In PD, the monoamine hypothesis is also unable to adequately explain the low rate of responders and the delayed mood improvement after antidepressant administration in responders. Moreover, it cannot sufficiently explain the complex manifestations of major depressive disorder. For instance, the relationship between PD severity and major depressive disorder follows a bimodal distribution curve rather than being linear: the first peak is more common during the early stages and the second one during the advanced stages of the disease. This suggests a potential reactive mechanism in response to the initial diagnosis and to the later a loss of functional independence secondary to physical deterioration (Cummings, 1992; Veazey et al., 2005).

## Treatment Strategy Based on the Biochemical Model

The most widely used strategy for the treatment of PD-dep is based on the biochemical model**.** Antidepressant medication remains as the mainstay in the treatment of PD-dep. Agents used to increase monoamine levels have been long time considered as the first line choice of PD-dep treatment. However, antidepressants seem to be less effective than expected or have intolerable adverse effects in many PD patients with major depresive disorder, including worsening of some motor symptoms, visual hallucinations, delusions and orthostatic hypotension, as well as several important drug interactions (Veazey et al., 2005; Wensel et al., 2005). Although selective serotonin reuptake inhibitors (i.e., SSRIs) are commonly prescribed to treat PD-dep, there is insufficient evidence to conclude on their efficacy, according to an evidence-based medicine review (Seppi et al., 2011). Additionally, SSRIs may have an antagonistic effect on dopamine, sometimes leading to a worsening of motor symptoms (Wensel et al., 2005). As already mentioned above, another pharmacological strategy involves the administration of D2/D3 receptor agonists (Barone et al., 2010; Seppi et al., 2011). However, their efficacy is questionable because of inconsistent results (Liu et al., 2013). The efficacy of other antidepressants such as tricyclic agents has not been definitely established but based on actual evidence, they are considered as being “likely efficacious” (Seppi et al., 2011).

For all these reasons, non-pharmacological strategies are mandatory in the treatment of PD-dep either to replace or to potentiate the beneficial effects of drugs.

## The Network Model of Depression in PD

Although the biochemical hypothesis for depression has dominated the scientific opinion for decades (Schildkraut, 1965), an alternative hypothesis has been recently proposed: the network hypothesis. According to this model, mood disorders reflect, at least in part, maladaptive neuronal plasticity in mood relevant networks, namely the inability of cortico-limbic circuits—whose key nodes are the prefrontal cortex, the amygdala and the hippocampus—to appropriately adjust their microstructure and function to some environmental experiences (Castrén, 2005, 2013). According to the network hypothesis, problems in information processing within neural networks, rather than changes in chemical balance, might underlie major depressive disorder. The therapeutic effect of antidepressant drugs might operate through a reactivation of an “early-life plasticity process”, that involves environment-driven recruitment of appropriate network connections (Castrén, 2005). Therefore, the way these networks activate and reorganize is dependent of the environment. This suggests that pharmacological treatments alone are not sufficient for improving mood and therefore require to be combined with appropriate rehabilitation, such as cognitive and behavioral therapy (CBT), that guides the plastic networks (Castrén, 2013).

## Treatment Strategy Based on the Network Model of Depression

**Figure 1:**
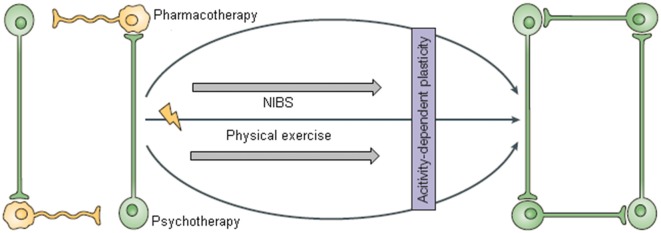
**This figure was adapted from Castrén (2005).** The network model of depression suggests that pharmacological treatments alone are not sufficient for improving mood and need to be combined with appropriate (neuro)rehabilitation to support the reorganization of plastic networks. With the permission of Nature Publishing Group (http://www.nature.com/npg_/index_npg.html).

**Figure 2:**
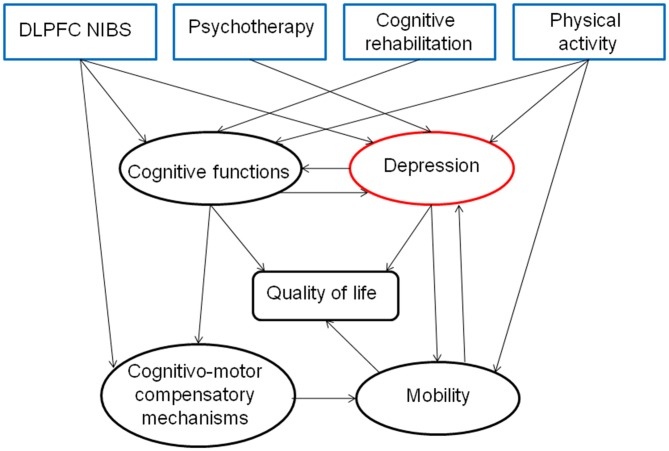
**Hypothesis about the direct and indirect effects of a combined therapeutic strategy for depression, highlighting the interest of a multidisciplinary approach for treating PD patients**.

We hypothesize here that efficient environment-driven network reorganization could be achieved by combining three kind of interventions: non-invasive brain stimulation (NIBS), CBT and physical training (Krystal, 2007; Castrén and Rantamäki, 2010; Figures 1, 2).

### NIBS

Electrical brain stimulation has a long history in the treatment of refractory depression. Electroconvulsive therapy (ECT) was introduced as a powerful therapeutic option for severe psychiatric disorders about 70 years ago. Since the early 50’s, ECT has been widely used with antidepressant purposes and is still considered as the most effective acute treatment for major depressive disorder (Kho et al., 2003; standardized effect size: −0.91, UK ECT Review Group, 2003). ECT is a procedure in which administration of an electrical current through the brain of anesthetized patients induces a generalized seizure that produces changes in brain functions, especially mood, by acting over frontotemporal circuits (Anderson and Fergusson, 2013). However, ECT has some important adverse effects such asystolia, tachycardia, delirium, agitation, memory impairment and transient neurological motor deficit which limit its usage.

Transcranial magnetic stimulation (TMS) has been touted as a safe alternative to ECT. TMS is a non-invasive technique that uses electromagnetic induction to generate weak electric currents in the stimulated cortex, modifying cortical activity (Rossi et al., 2009). Repetitive TMS (rTMS) is capable of producing a long-lasting modulation of cortical activity. From a neurobiological point of view, rTMS has been proposed to promote synaptic plasticity, an effect described as long term potentiation (LTP) or depression LTD. The effects of rTMS propagate beyond the stimulation site to impact a widely distributed set of connected brain regions (i.e., a brain network). Evidence suggests that these modulations are relevant in the therapeutic response (Lefaucheur et al., 2014).

In patients with major depressive disorder, many rTMS studies have been designed to stimulate the left prefrontal cortex with high-frequency (i.e., >1 Hz) stimulation during several daily sessions to achieve a long-lasting therapeutic effect. The rationale for the use of lateralized rTMS is based on the hypothesis that mood disorders may result from a relative imbalance between left and right frontal lobe activity (Davidson et al., 2002). According to this hypothesis, major depressive disorder is associated with a decreased activation of the left frontal lobe, which is normally involved in approach-related, appetitive behaviors. This imbalance between left and right frontal lobe functions would negatively impact onto prefrontal-limbic networks, composed of interconnected brain areas including the anterior cingulate cortex (ACC), the amygdala and the hippocampus (Bennett, 2011). The lateralized hypothesis of depression is supported by lesional and neuroimaging studies. Indeed, depressive symptoms are more frequent in left brain-damaged patients (Gainotti, 1972). More recent studies showed hypofunctioning left prefrontal cortex in people with major depressive disorder (Fitzgerald et al., 2006; Steele et al., 2007). High frequency rTMS over the left dorsolateral prefrontal cortex (DLPFC) can also influence distant brain regions that are compromised in major depressive disorder suggesting that its therapeutic effect may operate by modulating functional connectivity in broad cortical networks, notably the default mode and the central executive networks (Liston et al., 2014).

A recent meta-review of 15 meta-analyses concluded that DLPFC rTMS is an effective and safe brain stimulation technique for the treatment of medication refractory depression, with moderate to large effect size (i.e., weighted mean effect size from 0.39 to 0.89; Dell’osso et al., 2011). High frequency rTMS is now approved by the Food and Drug Administration (FDA) “to treat the symptoms of major depressive disorder without inducing seizure in patients who have failed at least one antidepressant medication and are currently not on any antidepressant therapy”^1^. The response rate is approximately 50% (Connolly et al., 2012) and has been reported to remain stable even after 6 months (Concerto et al., 2015). One limitation of rTMS in major depressive disorder is the inability to identify the optimal stimulation site in the left DLPFC. It is ususally recommended to stimulate a spot 5 cm anterior to the motor cortex along the curvature of the scalp (i.e., O’Reardon et al., 2007). It is not surprising that this technique leads to variability in both the stimulated region and therapeutic response (Herwig et al., 2001; Fitzgerald, 2009; Herbsman et al., 2009). Recently, new targets are being investigated. Four new potential regions have been identified as candidates for rTMS in major depressive disorder: dorsomedial prefrontal cortex (DMPFC), frontopolar cortex (FPC), ventromedial prefrontal cortex (VMPFC), and ventrolateral prefrontal cortex (VLPFC; Downar and Daskalakis, 2013). The DMPFC is perhaps the more promising target because of the link between lesions of this region and severe depression. In addition, a pioneer study has already shown the potential effectiveness of stimulating DMPFC for treating major depressive disorder (Bakker et al., 2015).

Moreover, the evidence that major depressive disorder is associated with an imbalance between hemisphere functioning is exclusively correlative and results are not always consistent across studies (Fitzgerald et al., 2006). A causal relationship between laterality and major depressive disorder is partially supported by previous TMS studies evaluating the effects of left and right rTMS (Chen et al., 2013). However, these studies compared high-frequency left to low-frequency right rTMS. Therefore, laterality is a factor that should be addressed, especially by comparing the effect of different rTMS protocols (inhibitory-excitatory/Left-right DLPFC) on clinical and brain responses in major depressive disorder.

In PD, NIBS has first been tested on motor symptoms. Indeed, when applied over the primary motor cortex, both rTMS and transcranial direct current stimulation (tDCS) are able to improve motor functions, as measured by the Unified PD Rating Scale (UPDRS-III; Broeder et al., 2015; Wagle Shukla et al., 2015).

rTMS has been claimed to be effective on improving PD-dep when applied over the left DLPFC (Fregni et al., 2004; Epstein et al., 2007). Fregni et al. (2004) reported a 41%-proportion of treatment-responders (with 50% reduction in the Hamilton Rating scale) with 15 Hz-rTMS for 10 days during 2 weeks. A randomized double-blind controlled trial using 5 Hz left DLPFC rTMS for 10 days in patients with PD-dep showed a significant decrease of 44% in the Beck Depression Inventory score lasting at least 30 days after the end of the stimulation protocol (Pal et al., 2010). A recent meta-analysis evaluating the effect of DLPFC rTMS on PD-dep has reaveled a significant improvement in the Hamilton Depression Rating Scale (HDRS) compared with sham-rTMS (standard mean difference = −0.88) and suggested a similar antidepressant efficacy as the one obtained with SSRIs (Xie et al., 2015).

### CBT

Another environment-driven approach for the treatment of major depressive disorder is CBT. This method was first developed by Beck’s (1995) and is based on a cognitive model of the relationship between how we think (cognition), how we feel (emotion) and how we act (behavior). Beck et al. (1979) proposed that disturbances in information processing are key features in major depressive disorder. According to this model, people with major depressive disorder hold negative core beliefs developed during childhood, which are re-activated by life events and produce automatically generated thoughts about the self, the world and the future. Therefore, CBT consists in helping patients to evaluate their thinking in a more realistic and adaptive way in order to modify negative automatic thoughts (Beck et al., 1979). CBT includes three main phases: a psychoeducation phase, a behavioral activation phase and a cognitive restructuring phase. The psychoeducation phase is related to teaching the cognitive model and understanding the dysfunctional information processing in major depressive disorder. After acceptance of the model and after having reviewed daily behaviors, proposals for planning new and rewarding activities are suggested. The psychotherapist and patients can afterwards work together in the identification of negative automatic thoughts that maintain the patient in the spiral of depression in order to better change them into more balanced and realistic thoughts. Therefore, CBT for major depressive disorder could be seen as a cognitive training for the learning of new skills regarding information processing. CBT is effective in treating major depressive disorder in adults (Hofmann et al., 2013), with a medium effect size of −0.69 to −0.42 (Cohen’s *d*) after correction for publication bias (Cuijpers et al., 2010). A meta-analysis reported a response rate between 57 and 87% in major depressive disorder (Leichsenring, 2001).

The beneficial effects of CBT in major depressive disorder have been associated with neuroplastic changes in relevant brain networks. (Goldapple et al., 2004; Dichter et al., 2010; Ritchey et al., 2011; Yoshimura et al., 2014). For instance, Ritchey et al. (2011), using functional magnetic resonance imaging (fMRI), showed that one weekly session of CBT during 30.3 weeks in average, led to an increased activity in the prefrontal cortex of depressed patients during emotion processing. In another study, Goldapple et al. (2004) showed cerebral glucose uptake changes in the cortico-limbic pathway, notably an increased metabolism in the hippocampus and a decreased metabolism in the DLPFC following CBT. The effects on brain structure and function have also been reported after CBT interventions in other psychiatric conditions. In a study involving people with chronic fatigue syndrome, de Lange et al. (2008) reported increased gray matter volume bilaterally in the DLPFC after approximately 16 sessions of therapy. Thus, an individualized tailored psycho-cognitive training approach such as cognitive restructuring could induce substantial and stable brain changes through neuroplastic mechanisms finally resulting in emotional and behavioral improvements.

CBT has received little scientific attention in PD patients. Only a few studies examined the effectiveness of CBT for PD-dep (e.g., Farabaugh et al., 2010; Dobkin et al., 2011; Yang et al., 2012). In a ten-session randomized controlled trial of CBT for PD-dep that included some patients taking antidepressants, symptom severity improved by 56% relative to 8% for the control group (Dobkin et al., 2011). Additionally, there were significantly more responders in the CBT group (i.e., number needed to treat 2.1; absolute risk reduction 48%), and large effect sizes were observed on all depression outcome measures.

Only one study examined the effect of psychological intervention (i.e., Mindfulness based intervention) on brain plasticty in PD patients (Pickut et al., 2013). Mindfulness based intervention is considered to belong to the third wave of CBT with the core goal of developing metacognitive awareness which is the ability to experience cognitions and emotions as mental events that pass through the mind with a compassionate non-judgmental stance. Therefore, mindfulness could be viewed as a kind of attention training (Jha et al., 2007). After mindfulness-based intervention, there was an increased gray matter density in key limbic regions, i.e., the amygdala and hippocampus. This finding suggests that psychotherapeutic interventions in PD patients have also the potential to influence cortical plasticity.

### Physical Exercise

According to the network hypothesis of depression, physical exercise can be viewed as an environmental stimulation adjuvant to CBT and NIBS, that guides efficient neural network reorganization (Figure 1).

The indication of regular physical activity as a therapeutic strategy in PD-dep is mainly supported by the positive effects observed in depressed people without PD. A meta-analysis examined the effects of exercise on depression/depressive symptoms in 58 randomized controlled trials (*n* = 2982). An overall effect size of −0.80 (Hedges’ g) indicated that participants in the exercise treatment group had significantly lower depression scores than those receiving the control treatment or no-treatment (Rethorst et al., 2009). The same authors also analyzed the moderating variables of exercise programs (i.e., duration, exercise type, frequency and intensity). Programs lasting 10–16 weeks resulted in stronger effects than shorter interventions. The exercise programs that combined erobic and resistance exercises resulted in larger effects than erobic or resistance training alone. It is noteworthy that converging evidence support the beneficial effect of physical activity on anxiety, which is frequently associated with depression (Wipfli et al., 2008). In addition, a few studies showed that a tailored exercise program is an effective way to improve performance of daily activities and both motor and non-motor symptoms, such as mood, in PD patients (i.e., decrease in Beck Depression Inventory-II score; Cugusi et al., 2014, 2015; Lattari et al., 2014).

Several hypotheses that involve biochemical, physiological, and psychosocial mechanisms have been offered to account for the effect of exercise on major depressive disorder. At a psychological level, physical exercise can increase perception and awareness of the body and physical self, with a positive impact on self-esteem and therefore fewer feelings of depression (Knapen et al., 2005; Eriksson and Gard, 2011). At a neurobiological level, erobic exercise triggers plasticity-related changes in the brain, such as synaptogenesis, enhanced glucose utilization and neurogenesis (Speelman et al., 2011). In PD, erobic exercise may be beneficial in the improvement of motor, cognitive and emotional functioning by enhancing neuroplasticity and reducing neuroinflammation (Speelman et al., 2011).

## Towards a Multidimensional Approach for Treating PD-dep

Emotion, movement and cognition are linked and are mediated through fronto-striatal loops. The prefrontal cortex is a central cerebral region that provides top-down regulation of attention, inhibition, motivation, and emotion through connections with subcortical structures (Tekin and Cummings, 2002). There are three main regions involved in major depressive disorder situated in the anterior part of the frontal lobe: the DLPFC, the orbritofrontal cortex (OFC) and the ACC. These cortical areas belong to three distinct fronto-striatal circuits which mediate cognitive functioning, motivation and emotion regulation (Bonelli and Cummings, 2007). The DLPFC, which is a key region in major depressive disorder, is presumed to be mainly related to cognitive processing. Impaired DLPFC functioning can result in cognitive biases such as attention allocation toward negative stimuli and distorted information processing, but also more global cognitive deficits such as attention, working memory and executive dysfunctions (Murrough et al., 2011). According to Beck et al. (1979) cognitive model, distorted automatic cognitions negatively influence mood and behavior. Therefore, proper top-down regulation of emotion might be achieved through normalization of DLPFC functioning by combining CBT, cognitive training and NIBS.

Although medications and CBT, alone or in combination, are considered effective treatments for major depressive disorder, up to 30% of patients show no improvement or only a partial response. Previous reports support the efficacy and good tolerability of rTMS in the treatment of major depressive disorder (Horvath et al., 2010). So far however, no study has assessed the effect of combining brain stimulation with CBT in the treatment of PD-dep, even if both methods are able to induce changes in prefrontal activity (Cardoso et al., 2008; Ritchey et al., 2011). Only two case-report studies of feasibility have tested the effect of NIBS combined with CBT on patients with treatment-resistant major depressive disorder (Vedeniapin et al., 2010; D’Urso et al., 2013). D’Urso et al. (2013) applied daily tDCS, another NIBS technique, during 2 weeks in addition to CBT in a 52-year old woman with severe and chronic major depressive disorder. This synergistic treatment induced substantial improvement in the HDRS with complete remission of symptoms, which remained stable over 12 months. The other available report (Vedeniapin et al., 2010) examined the effect of CBT together with left prefrontal cortex rTMS on a 26 year old female student with resistant major depressive disorder. After a 14-week-treatment with 3 weekly rTMS sessions and a total of 14 CBT sessions, the patient showed reduction in symptoms with a remission that lasted up to 3 months after the end of the intervention. However, the precise contribution of each treatment cannot be separetely accounted. For this reason, 4-arm, randomized controlled trials are needed to test the hypothesis of a synergistic effect of nonpharmachological strategies in the treatment of PD-dep (Group 1: sham rTMS + placebo psychotherapy or waiting list condition; Group 2: rTMS + placebo psychotherapy or waiting list condition; Group 3: CBT + sham rTMS; Group 4: rTMS + CBT). A recent randomized double-blinded trial lead the way for future controlled studies, by combining cognitive training with simultaneous DLPFC tDCS in PD patients with mild cognitive impairments (Biundo et al., 2015).

## Conclusion

In summary, we reviewed here the importance of treating PD-dep because of its negative direct and indirect influence on quality of life and mobility. According to the network hypothesis of depression, we suggest a new type of combined therapy for treating PD-dep, focused on the optimization of activity-dependent plasticity in the prefrontal cortex, especially DLPFC rTMS and CBT (Figure 2). This approach could be first focused on behavioral and cognitive activation by practicing physical activity, enhancing social interactions and intellectual activities and secondly on cognitive restructuring of negative automatic thoughts. This kind of combined therapy might have additive and synergistic influences on mood through an increased cortico-limbic stimulation-induced plasticity.

## Author Contributions

KD did the literature search and wrote a first draft; GG corrected and revised the manuscript.

## Conflict of Interest Statement

The authors declare that the research was conducted in the absence of any commercial or financial relationships that could be construed as a potential conflict of interest.

## Footnotes

^1^http://www.accessdata.fda.gov/scripts/cdrh/cfdocs/cfcfr/cfrsearch.cfm?fr=882.5805
